# Novel *in vitro* and mathematical models for the prediction of chemical toxicity

**DOI:** 10.1039/c2tx20031g

**Published:** 2012-09-05

**Authors:** Dominic P. Williams, Rebecca Shipley, Marianne J. Ellis, Steve Webb, John Ward, Iain Gardner, Stuart Creton

**Affiliations:** a MRC Centre for Drug Safety Science , Department of Molecular and Clinical Pharmacology , Institute of Translational Medicine , The University of Liverpool , Sherrington Building , Ashton St. , Liverpool , L69 3GE , UK . Email: dom@liv.ac.uk ; Fax: +44 (0)151 794 5540 ; Tel: +44 (0)151 794 5791; b Department of Mechanical Engineering , University College London , Torrington Place , London WC1E 7JE , UK; c Department of Chemical Engineering , University of Bath , Claverton Down , Bath , BA2 7AY , UK; d Department of Mathematics and Statistics , University of Strathclyde , Livingstone Tower , 26 Richmond Street , Glasgow , G1 1XH , UK; e School of Mathematical Sciences , Loughborough University , Loughborough , LE11 3TU , UK; f Simcyp Limited , Blades Enterprise Centre , John Street , Sheffield S2 4SU , UK; g NC3Rs Gibbs Building , 215 Euston Road , London , NW1 2BE , UK

## Abstract

The focus of much scientific and medical research is directed towards understanding the disease process and defining therapeutic intervention strategies. The scientific basis of drug safety is very complex and currently remains poorly understood, despite the fact that adverse drug reactions (ADRs) are a major health concern and a serious impediment to development of new medicines. Toxicity issues account for ∼21% drug attrition during drug development and safety testing strategies require considerable animal use. Mechanistic relationships between drug plasma levels and molecular/cellular events that culminate in whole organ toxicity underpins development of novel safety assessment strategies. Current *in vitro* test systems are poorly predictive of toxicity of chemicals entering the systemic circulation, particularly to the liver. Such systems fall short because of (1) the physiological gap between cells currently used and human hepatocytes existing in their native state, (2) the lack of physiological integration with other cells/systems within organs, required to amplify the initial toxicological lesion into overt toxicity, (3) the inability to assess how low level cell damage induced by chemicals may develop into overt organ toxicity in a minority of patients, (4) lack of consideration of systemic effects. Reproduction of centrilobular and periportal hepatocyte phenotypes in *in vitro* culture is crucial for sensitive detection of cellular stress. Hepatocyte metabolism/phenotype is dependent on cell position along the liver lobule, with corresponding differences in exposure to substrate, oxygen and hormone gradients. Application of bioartificial liver (BAL) technology can encompass *in vitro* predictive toxicity testing with enhanced sensitivity and improved mechanistic understanding. Combining this technology with mechanistic mathematical models describing intracellular metabolism, fluid-flow, substrate, hormone and nutrient distribution provides the opportunity to design the BAL specifically to mimic the *in vivo* scenario. Such mathematical models enable theoretical hypothesis testing, will inform the design of *in vitro* experiments, and will enable both refinement and reduction of *in vivo* animal trials. In this way, development of novel mathematical modelling tools will help to focus and direct *in vitro* and *in vivo* research, and can be used as a framework for other areas of drug safety science.

## Abstract 2

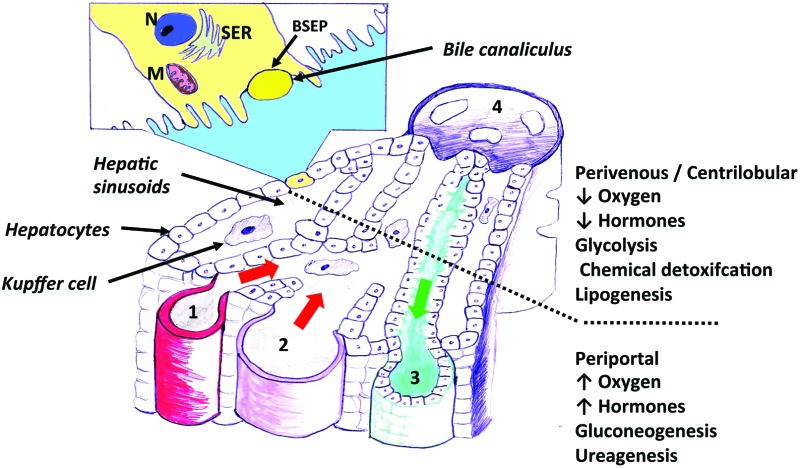
Reproduction of centrilobular and periportal hepatocyte phenotypes in vitro is crucial for sensitive detection of cellular stress, combined with mathematical modelling, is a novel tool for drug safety research.

## 1. Introduction to adverse drug reactions and chemical toxicity

### 1a. Adverse drug reactions

Adverse drugs reactions (ADRs) are a major cause of drug attrition and are a huge burden on healthcare systems. In the US, ADRs rank between the 4th and 6th leading cause of death.^1^ The majority of ADRs are predictable, and become apparent during preclinical and clinical toxicity assessment,^2^ and those that manifest in the human population are commonly as a result of a prescribing error or through drug–drug interactions (DDIs^3^). As a consequence of evidence of toxicity, 16 out of 548 (2.9%) new chemical entities that were approved for the US market between 1975 and 1999 were subsequently withdrawn from the market, and 56 out of 548 (10.2%) acquired a black box warning.^4^ Excessive dose, drug accumulation and/or the formation of chemically reactive metabolites (CRMs) have been implicated in many off-target (including idiosyncratic) ADRs. Unlike these ‘predictable’ types of ADRs, idiosyncratic ADRs are rarer but are more likely to be fatal.^3^ Idiosyncratic ADRs are often detected only when the offending drug has been released into the wider population following regulatory approval. Idiosyncratic ADRs are generally immune mediated and include hepatotoxicity, severe cutaneous reactions, anaphylaxis and blood dyscrasias.^5,6^

The organ that is most frequently affected by CRM-mediated ADRs is the liver. Drug-induced liver failure (DILI) poses a major clinical issue and has become the leading cause of acute liver failure and transplantation in Western countries.^7,8^ DILI accounts for more than half the cases of acute liver failure in the United States. DILI also represents a major challenge for industry and regulatory authorities: it is a leading cause for termination of further substance development in preclinical and clinical phases, and it is also the most common single adverse drug reaction leading to refusal of market approval. However, in many instances a drug's hepatotoxic potential is only recognized post-marketing and DILI has therefore also been a significant reason for withdrawing drugs from the market. Acetaminophen (paracetamol; APAP) is responsible for 80% of drug-associated cases of liver failure.^8^ APAP-induced hepatotoxicity is generally predictable from our understanding of its metabolism; however, many other drugs cause idiosyncratic DILI, which, although rare and unpredictable, can cause significant morbidity and mortality. Studies with model compounds and drugs — such as APAP — have helped to define the roles that chemical stress and drug bioactivation have in the various biological outcomes that may be triggered by CRMs. These include effects on transcription factors and/or signalling protein-adaptation (cell defence), apoptosis, necrosis, inflammation and activation of the innate and adaptive immune systems.^9^

Many different classes of drugs licensed for clinical use are known to cause DILI in man, and the cumulative DILI burden on professional health services coupled with patient well-being and/or mortality is high. Adverse drug reactions affecting the liver may be classified in two main types:

• Dose-dependent DILI which can be replicated readily in various animal species and is evident during preclinical safety testing.

• Human-specific DILI, which cannot be predicted in animal models or animal systems, and include idiosyncratic drug reactions (drug- or individual (Human Leukocyte Antigen (HLA) restriction)-specific) are unpredictable, occur only in certain susceptible patients and have a complex dose-dependent relationship.

Over 1000 drugs and herbal products have been associated with idiosyncratic hepatotoxicity,^10–12^ which is responsible for more than 10% of all cases of acute liver failure in the US. The incidence of idiosyncratic DILI caused by some drugs can be high as 1 in 100 patients (*e.g.*chlorpromazine), but more typically lower at 1 in 10 000 patients (*e.g.*flucloxacillin). Idiosyncratic DILI is of major concern because it is not predictable from pre-clinical safety assessment studies due to a lack of predictive models, and consequently is rarely evident until late clinical trials or after regulatory approval.

Despite extensive work over the past 20 years, no new non-clinical tests and/or clinical biomarkers have been forthcoming for DILI that have been universally accepted by drug-developers, clinicians and regulators.^13^ *In vivo* safety testing in pre-clinical species ensures that drugs, which enter clinical trials, do not cause reproducible and dose-dependent liver injury. However such test systems have failed to predict some serious cases of DILI that actually occurred in man, in particular those of an idiosyncratic nature. An improved understanding of mechanisms that underlie DILI in man is therefore required to enable design of drugs that have minimal potential to cause this adverse reaction in the patient population.

There are a number of different mechanisms through which drugs and their metabolites have been shown to cause ADRs. These include mitochondrial injury, transporter interactions, innate and adaptive immune system activation, direct cytotoxicity and phospholipid/lysosome liabilities. It is beyond the scope of this review to discuss novel *in vitro* and mathematical models for each of these toxicity mechanisms. The development of more sensitive *in vitro* models that replicate the *in vivo* environment allows re-investigation of drugs that have caused serious injury in man or adverse signals in experimental animals. Additionally, closer recapitulation of *in vivo* conditions would naturally encompass wider mechanistic coverage. These biological signals may have been overlooked in previous investigations due to a lack of appropriate technology or information. More effective *in vitro* systems would allow pragmatic compound ranking during drug discovery, aiding *in vitro*/*in vivo* translation with species/dose selection and translation to man. Therefore, we will focus on novel models exploring metabolic liabilities of drugs and chemicals. In particular, the design of *in vitro* systems that more closely mimic the *in vivo* environment, especially with respect to metabolism and bioactivation, and how these systems can be mathematically modelled.

The concept that small organic molecules can undergo bioactivation to electrophiles and free radicals, and elicit toxicity by covalent modification of cellular macromolecules, has its basis in chemical carcinogenicity and the pioneering work of the Millers^14–16^ (who studied the hepatotoxic effects of *p*-dimethylaminoazobenzene in the rat and found that aminoazo dyes become tightly bound to the protein constituents of liver tissue). The application of such concepts to human drug-induced hepatotoxicity was established through the studies of Brodie, Gillette, and Mitchell on the covalent binding to hepatic proteins of toxic doses (samples were obtained from overdose patients) of the widely used analgesic APAP. This concept is now well established, and there is a wealth of evidence to suggest that drug metabolism to a Chemically Reactive Metabolite (CRM; bioactivation) is the initiating step in a number of direct and immune mediated toxicities.^5,17^ A common mechanism for the detoxification of CRM occurs *via*glutathione (GSH) conjugation.^18,19^ The identification of drug metabolite-GSH adducts in a preclinical setting is sometimes treated as a hazard signal as it is indicative of CRM formation; conversely it could be argued that a GSH adduct is an indication of an effective detoxification system. Idiosyncratic ADRs are not reproducible in preclinical species, and so, recently there has been an increased focus on the development of surrogate endpoints of toxicity (biomarkers; either biological or chemical), animal models and improved understanding of structure-activity relationships of drugs. A list of chemical moieties frequently associated with drug bioactivation and subsequent toxicity (structural alerts) has been compiled.^17^ One approach to improving a drug's safety profile would be to avoid incorporating these structural alerts altogether; however, these moieties often are key to the pharmacology of the drug. In addition, the presence of a structural alert does not automatically result in a toxic compound; not all drugs that contain a structural alert are bioactivated and not all bioactivated drugs are toxic.^17^ The structure-activity relationships are, therefore, not straight forward.

### 1b. Requirement for improved models of hepatotoxicity

The current *in vitro* test systems used by the pharmaceutical industry include simple liver-derived cell-based or sub-cellular models that are poorly predictive of toxicological potential. Importantly, such models do not take account of the mechanistic basis of human DILI or the environmental conditions under which human DILI might occur. Critically, there has not been a concerted effort at harmonisation of current, emerging and novel test systems, or the strategies for their implementation, across the Pharmaceutical Industry. As a result, too little is understood about how the current test systems compare physiologically with human liver, what are the critical signalling systems, and the mechanisms by which DILI occurs in man, to be able to produce more predictive test systems.

It has been estimated that a 10% improvement in predicting failure before the initiation of expensive and time-consuming clinical trials could save upwards of $100 million in the costs associated with drug development.^20^ The need for better predictive models for DILI is clear and obvious. DILI is widely regarded both during clinical development and post-approval as a leading cause of:

• Drug attrition due to preclinical toxicity

• Drug attrition due to toxicity in man in late clinical trials

• Drug withdrawal post-licensing

• Cautionary and restrictive labelling

• Failed regulatory drug registration

• Serious illness in man

Therefore, it constitutes a major safety concern for drug development that severely impacts the profit margins of pharmaceutical companies through increased costs, longer development times and reduced market capitalization. Elimination of drug candidates likely to cause hepatotoxicity at early stages of drug discovery could significantly decrease the rate of attrition and cut the cost of drug development. In the context of prediction of DILI there are numerous issues to solve. Presently, there is a lack of sufficiently organized data to make an informed conclusion on the predictivity of non-clinical studies for identifying the risk of significant hepatotoxicity in clinical trials and in the post-marketing population. Additionally, there is limited predictive value of animal studies; some compounds have progressed into human trials and have then caused severe human toxicities, and no patterns existed in the animal studies that signalled these events. Currently, retrospective studies are being used to shed light on potential mechanisms that underlie toxicity. Ximelagatran, a first-generation thrombin inhibitor, was developed as an alternative to warfarin for individuals requiring anticoagulation therapy. However, up to 15% of patients treated with this drug experienced elevations in serum ALT levels, which were severe and life threatening in some cases.^21,22^ An association between the HLA class II allele HLA-DRB1*07 and elevations in serum ALT levels was observed in 74 ximelagatran treated patients, compared with 130 controls who were also given ximelagatran but who did not develop DILI; these findings were replicated in an additional dataset.^21,22^ Neither preclinical nor clinical studies of ximelagatran had suggested an immune-mediated mechanism underlying the increase in serum ALT levels associated with the use of this agent. However, studies in guinea pigs had suggested the possibility of skin sensitization in response to ximelagatran and some individuals exposed to ximelagatran during the manufacturing process developed a skin rash. Additionally, ximelagatran and its metabolites were shown to selectively inhibit the binding of peptides to HLA-DRB1*0701, further supporting a role for immune dysregulation in the pathogenesis of this instance of DILI.^21,22^ This exemplifies the fact that human-specific and idiosyncratic hepatotoxicity cannot be detected by conventional animal toxicity studies, with there being no suitable preclinical models combined with a poorly understood pathogenesis. It is likely to arise from complex interactions among genetic, non-genetic host susceptibility, and environmental factors. Additionally, interspecies differences in bioavailability, distribution and metabolism may explain a number of false positives and false negatives.

A much better understanding of the molecular mechanism(s) of liver toxicity in man, as well as the underlying reasons why non-clinical studies fail to prevent compounds which produce serious human hepatotoxicity from proceeding in the clinic, could result in the development of a more predictive non-clinical testing strategy. This is a critical prerequisite for any improvement in the detection of hepatotoxicity during the pre-clinical phase of drug development. Experimental approaches have focused on the development of various *in vitro* assays that can be used to assess *in vivo* effects. *In vitro* studies on liver cells have been developed to reduce or replace animal experiments. However, most of the *in vitro* tests in use are based on cell lines, which do not necessarily represent normal cell physiology.

The current *in vitro* test systems are poorly predictive of toxicological potential for a number of reasons. (1) The physiological gap between the cells that are currently used and human hepatocytes as they exist in their native state; (2) the lack of physiological integration with other cells and systems within the liver, that are required to amplify the initial toxicological lesion into overt toxicity, and; (3) there is no way to assess how low level cell damage induced by a drug may, in certain circumstances, lead to overt DILI in only a small minority of patients (*i.e.* idiosyncratic hepatotoxins). New approaches that improve upon conventional processes of risk assessment and safety evaluation are currently sought. Numerous promising new technologies and approaches have been described or are being developed which replicate many of the key biological processes implicated in reproducible and idiosyncratic/human-specific DILI. These range from simple cell systems to complex *in vivo* models, and may have the potential to enhance prediction and risk assessment of DILI in man if used during drug discovery and/or pre-clinical development. The choice of system or model to use depends upon the biology under investigation, for example in tissue engineering, it is widely accepted that 3D tissue culture models behave very differently to 2D models.

### 1c. Other disciplines to benefit from improved *in vitro* and mathematical models of toxicity

Global regulatory bodies require acute toxicity testing. The classification and labelling of chemicals based upon their hazardous properties is a major focus for these studies, whilst extending this information through to risk assessment and management is being attempted where possible. Although legislative controls around the world differ slightly with regard to their requirements and the broad hazard categories, the basic purpose of acute toxicity testing is the same: to allow substances to be categorised according to their potential hazards and the dose required to cause toxicity.^23^ Investigation of the potential to cause chemically-mediated toxicity is particularly important with legislation, such as the European Union REACH and Cosmetics regulations, and the Canadian Dangerous Substances List.^24^ In the EU, REACH is a regulation adopted to improve the protection of human health and the environment from the risks that can be posed by chemicals, while enhancing the competitiveness of the EU chemicals industry. It also promotes alternative methods for the hazard assessment of substances in order to reduce the number of tests on animals. In principle, REACH classification and labelling applies to all chemical substances, including industrial chemicals, biocides, active pesticide ingredients and final formulations, isolated pharmaceutical intermediates, new food additives, cosmetic ingredients, and consumer products. Therefore, the regulation has an impact on most companies across the EU. This legislation will help ensure that all chemicals manufactured or imported will have appropriate toxicology safety profiles.^23,24^ REACH became law in June 2007. It stipulates that all chemicals sold in the EU in annual quantities of more than one tonne (at least 30 000 compounds) must be registered, along with toxicity data, by 2018. When REACH was taking shape, it was clear that more stringent chemical-safety testing would require many more tests on laboratory animals. This has raised a number of deep concerns, principally ethical, but also including the financial costs, or even whether there were enough laboratories to conduct all the tests that would be required.^25^

Clearly, gathering all toxicological information utilizing animal testing would be costly and carries a significant ethical responsibility. It is thought that a thorough gap-analysis investigation of the available toxicological data will highlight areas of data-deficiency, and rather than conducting further animal testing, this will enhance the development and validation of emerging and novel techniques, technologies and platforms, expanding the chemical and biological knowledge base and allowing improved prediction of safety. Ultimately this will enable platforms to be defined to enhance knowledge depth, as opposed to purely being used for testing. The two main principles underlying this approach are (1) that similar chemicals should elicit similar downstream toxicological profiles and (2) both unbiased and case-study approaches are required to address this issue.^26–29^ Therefore, chemicals that have well characterised pharmacokinetic, metabolic and toxicological profiles can be placed into specific categories, either based upon similarity profiles across both chemistry and nature of the downstream biological perturbation elicited. Subsequently, chemicals with less data available (*e.g.* only *in vitro* data) can be assessed for hazard to man using read-across processes to fill data deficits in the toxicology allowing enhanced prediction of safety.^27,30^ This approach has been shown to be of use to predict a range of chemicals and toxicological endpoints, such as skin and respiratory sensitization, mutagenicity and carcinogenicity, reproductive toxicity, toxicity of petroleum derivatives, nickel compounds, repeat dose toxicity and acute fish toxicity, excellently reviewed by the Cronin lab.^24,31–34^ However, in order to address these data deficient areas we need more innovative *in vitro* and *in silico* approaches that take into account the mechanistic basis underlying the perturbation of normal biological processes by drugs and chemicals **– *in man***.

## 2. Physiological replication in *in vitro* models of hepatotoxicity

**Fig. 1:**
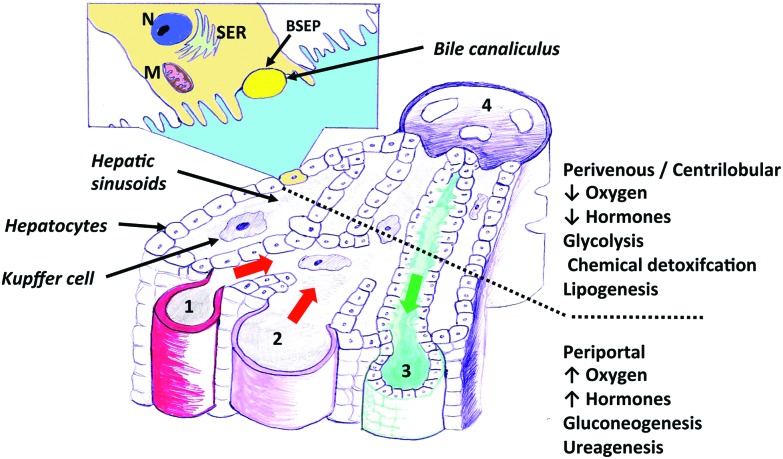
A diagram illustrating the functional units of an hepatic acinus within liver tissue. An enlarged view of an hepatic acinus containing the portal triad – hepatic arteriole (1), portal vein (2), bile duct (3), central vein (4) and the direction of bile (green arrow) and blood (red arrow) flow. Inset. A simplified look at how the hepatic acini individually contribute to the drainage system within liver tissue, which is the unit supplied and drained by terminal branches of portal triad vessels. Nucleus (N), Smooth endoplasmic reticulum (SER), Bile salt export pump (BSEP), Mitochondrion (M).

Current *in vitro* models offer both a relatively homogeneous view of liver function and cellular phenotype, when in reality, the morphology and function of hepatocytes vary enormously with their position along the liver sinusoids from the portal triad to the central vein. This phenomenon, termed zonation, has been described in practically all areas of liver function^35^ (Fig. 1). Bioenergetic processes, carbohydrate-, lipid-, nitrogen- and xenobiotic metabolism, bile acid conjugation and detoxification processes, have all been predominantly located within separate hepatic zones. The distribution of function along the length of the sinusoid is thought to be regulated by diverse factors such as oxygen and hormone gradients, nutrients and matrix composition. Importantly, the effects of the aforementioned factors upon non-parenchymal cell distribution and signalling, may alter the cross-talk between these cells and hepatocytes, assisting in defining the ultimate phenotype of a hepatocyte at a particular sinusoidal position. The regional characterization of hepatic responses to model hepatotoxins is well described, especially with compounds such as APAP, which elicits centrilobular hydropic, degenerative necrosis in the centrilobular zones of rodent and human liver. This necrotic pattern is also well characterised for carbon tetrachloride and bromobenzene in rodents. Less well characterised are the molecular processes leading to subsequent proliferation in periportal and midzonal regions in rodents.^36^ The regional hepatic injury pattern observed after administration of methapyrilene and allyl alcohol to rats, consists of periportal necrosis.^36,37^ Importantly, differential zonal responses to hepatotoxins, assists in dissecting more sensitive mechanistic details, which are highly unlikely to be observed in *in vitro* models. For example, the periportal rat hepatotoxin, methapyrilene, elicits initial glutathione depletion in periportal hepatocytes, whilst stimulating glutathione synthesis and adaptation in centrilobular hepatocytes. In *in vitro* hepatocyte incubations with methapyrilene, this is simply manifest as glutathione depletion and necrosis, with important adaptive responses being missed.^37^ It has been observed with the centrilobular hepatotoxin, APAP, using immunohistochemical techniques that there were dynamic changes in the lobule zonation of glutathionylated proteins.^38^ At 1 h after APAP exposure, the level of glutathionylation decreased in a single layer of hepatocytes around the central veins but increased in the remaining centrilobular hepatocytes.^38^ The increase correlated with the immunohistochemical localization of APAP covalently bound to protein.^38^ Subsequently, the level of glutathionylation decreased over time in the centrilobular regions. These temporal and zonal pattern changes in protein glutathionylation after APAP exposure indicate that protein glutathionylation may play a role in protein homeostasis during APAP-induced hepatocellular injury.^38^

## 3. Tissue engineering in bioartificial livers and hepatic bioreactors

In an attempt to replace the use of animal-derived tissue or *in vivo* models, numerous bioreactor systems have been used to engineer liver tissue, to act as an *in vitro* liver model. In theory it is possible to replicate the liver environment by careful selection and control of the cell culture environment in the bioreactor. Providing the correct environment necessitates (a) accurate mass transfer mechanisms of chemicals to the cells and metabolites away from the cells; (b) appropriate physical conditions, *i.e.* temperature, pressure, concentration profiles, shear stresses, 2D or 3D cell anchorage; (c) appropriate biochemical conditions, *i.e.* cell-matrix interactions, cell population density, provision of endocrine signalling molecules. All these factors must be at a size and with dimensions that allow accurate replication of liver function, both chemically and spatially, that is zonation ideally needs to be achieved. In the liver, plasma flows through the epithelial fenestrations and across the space of disse to the hepatocytes; as such, hepatocytes are not in direct contact with the blood flow. It is essential to replicate the *in vivo* flow environment if physiological function is to be achieved in a bioreactor. This is for two key reasons: fluid shear stresses, and mass transport considerations. If media is in direct contact with the cells, fluid shear stresses will be exerted on those cells. Shear stress is known to have an effect on cell behaviour – it has been reported that media flow upregulates primary human hepatocyte detoxification gene expression^39^ (although these findings may be due to the concentrations experienced as opposed to shear alone). Secondly, nutrient (and chemical) delivery/removal to/from the cell population is a direct function of the flow environment, given that nutrients and chemicals are transported in a dissolved state in the blood plasma. This also has implications for scale-up of bioreactors to a larger size required for tissue engineering on a clinical scale, as increased flowrates will be required to obtain the same media concentration profile.

**Fig. 2:**
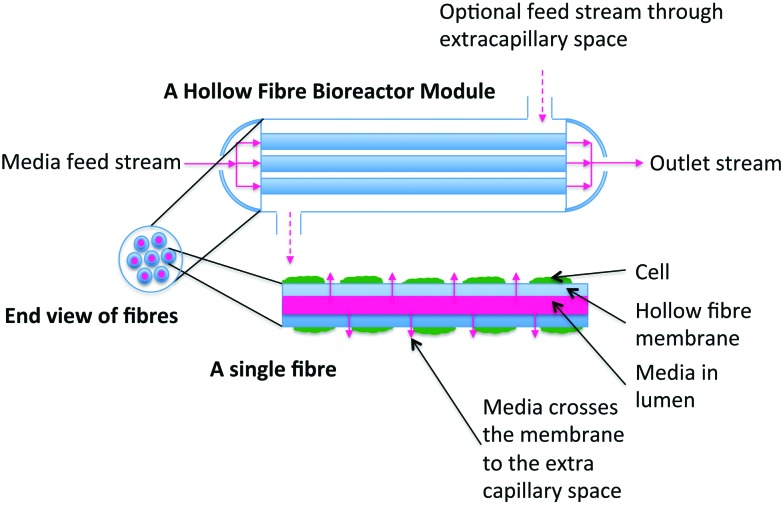
Sketch of a hollow fibre membrane bioreactor. The hollow fibre membrane acts as the blood vessel through which media is added, with cells cultured on or around the outside of the fibres. Ports on the sides of the module allow addition or removal of cells, media, and other components.

Bioreactors that have been used to date as *in vitro* liver models are summarised in Table 1; while they all have advantages and disadvantages, it is clear that hollow fibre membrane bioreactors (HFB) most closely represent the ideal design (see Fig. 2 for the setup of a HFB). Only membrane bioreactors separate hepatocytes from the main media supply (although the inner cells in spheroids are also removed from the flow and will receive media by diffusion) and thus enable the *in vivo* fluid shear environment to be replicated. In fact, shear can be applied independently, if desired, by side ports. These side ports can be used to supply different media types as well as apply shear, if desired and can be used to remove ‘bile’. Further, by controlling the media flowrates and pressures, the precise flow environment can be prescribed to mimic the nutrient and chemical delivery environment *in vivo*.

**Table 1:** Bioreactor designs used for *in vitro* liver models

Bioreactor design	Is there direct contact of cells and media?	Can a second feed be supplied to the cells?	Can shear experienced by the cells be controlled separately to the media stream?	Can zonation be induced?	Can ‘bile’ be collected separately to exit media?	Example reference for bioreactor design
*Ideal design*	*No*	*Yes*	*Yes*	*Yes*	*Yes*	
Hollow fibre membrane	No, except*	Yes	Yes	Yes	Yes	125–127*. See also^41^ main focus is BALs^128^
Rectangular slide (flat bed)	Yes	No	No	Yes	No	40
Macro-fabricated array bioreactor (flat bed)	Yes	No	No	Yes	No	43,44
Spheroids in a stirred tank	Yes	No	No	To an extent through the spheroid	No	46
Rotating HFB with cell-cytodex spheroids	No	No	Yes	To an extent through the spheroid	No	47
Flat sheet membrane	No	Yes	Yes	Yes	Yes	129–133
Lab-on-a-chip bioreactors ‘liver chip’ (flat bed)	Yes	No	No	Yes	No	42

A major consideration for a liver bioreactor is the replication of zonation. This was first achieved using a flat plate configuration;^40^ subsequent designs include the macro-fabricated array bioreactor^41^ (flat bed), and the lab-on-a-chip bioreactors ‘liver chip’ (flat bed) mentioned in the review by Lerapetritou and co-workers.^42^ The microfabricated flat plate bioreactors by Park and co-workers^43,44^ could also induce zonation, and do so linearly so that the waste is removed with the media outlet. Hollow fibre bioreactors build on this and their most significant benefit is the fact that they can be operated to replicate both zonation,^45^ and the sinusoid physiology. The hollow fibres, which are porous tubular membranes, act as pseudovascularisation. In the liver scenario each fibre acts as a sinusoid; given that the fibre walls are porous, media can flow across them to the cells, but importantly the cells are protected from the flow of the media in the lumen. This means the media supply rate can be altered without risk of shear damage to the cell population. It could be said that cells in spheroids, such as those recently reported^46,47^ will experience zonation, as far as they will experience a concentration gradient as media diffuses to the centre of the constructs, and waste diffuses back out. This does not however replicate the linearity of the sinusoid.

Hollow fibre bioreactors are not without their own challenges. Achieving homogeneous seeding is more complex than for a flat surface. Further, there are more components to deal with than flat sheet/bed bioreactors so the user needs additional training (and although experience shows that, with training, fabricating and operating them is possible by a wide range of users).

Hollow fibre bioreactors have a large surface area to volume ratio thereby making them space-efficient and more media efficient, both of which are important from an economic perspective particularly on a larger-scale beyond the lab bench, and as such they are used for a number of industrial applications. Hollow fibre bioreactor fluid dynamics and mass transfer are well characterised due to their use in a range of fields including waste water treatment, cheese making and hemodialysis, as well as mammalian cell culture including for bioartificial livers (BAL) which Wang 2010^41^ covers in a comprehensive review. A good introduction to hollow fibre membrane bioreactors can be found in Coulson and Richardson's Chemical Engineering Volume 2^48^ and elsewhere for more detailed design information.^49^

## 4. Mathematical modelling for bioartificial liver and hepatic bioreactor design

To enable broader and interdisciplinary applications of HFBs, it is essential to tailor the HFB operation to fulfill the requirements of the application under consideration. One route to achieve this is through mathematical models that describe the coupling between fluid and mass transport and the biochemical and biomechanical environment of the cells. It is essential that such models provide useable operating equations to allow accurate operation of the bioreactors by non-experts, and numerous studies in the literature have focused on such an approach.

**Fig. 3:**
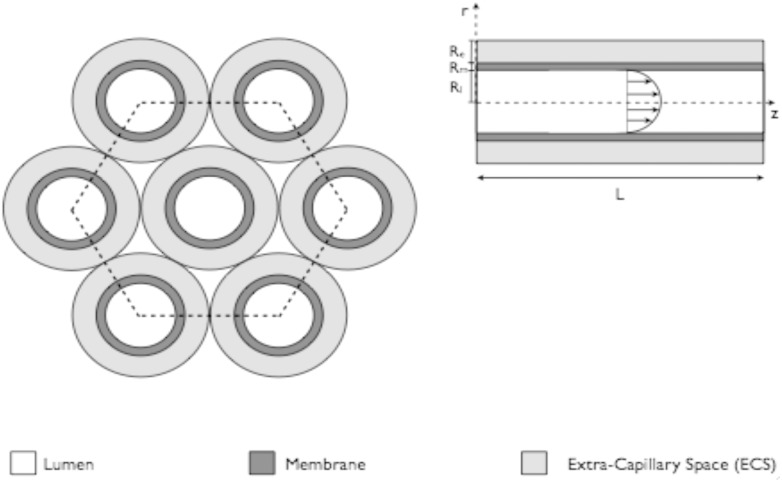
A bundle of HFB demonstrating the principle of Krogh cylinders. One fibre surrounded by an annulus of ECS can be used to describe the entire fibre bundle when interstitial spaces between the fibre are ignored. The length of an individual fibre is denoted L; the radius of the lumen is denoted R_l_, whilst the depth of the membrane and ECS spaces are denoted R_m_, R_e_, respectively.

The structure of a HFB is demonstrated in Fig. 2 and 3; it consists of a bundle of fibres housed in a bioreactor. Each fibre consists of a central cylindrical lumen surrounded by a porous membrane, then extra-capillary space (ECS). Cells can be seeded on the outer surface of the fibres (as relevant for hepatic considerations), or in a matrix surrounding the fibres, and media is driven through the fibre lumen under an applied pressure gradient. Given that the membrane is porous, nutrients, waste products, proteins and chemicals can permeate across it, to or from the cells. Further, exit ports in the ECS space may be opened to promote a fluid flow through the cellular space – this may be used to enhance nutrient delivery to the cell population, or to impose controlled fluid shear forces to the cells if required by the population under consideration. Mathematical models for mass transport in these systems are broadly divided into whether these ports are held shut (diffusion-limited) or open (convection-enhanced), according to the fluid flow regime that each scenario promotes. The general modelling frameworks take the form of convection–diffusion–reaction equations, where convection is applicable to the regions of the bioreactor with fluid flow, and the reaction component is used to, for example, describe uptake/production of nutrients/waste products by the cell population. Fluid flow is generally described using either Navier–Stokes or Darcy equations, depending on whether the flow is free (*e.g.* in the lumen), or through a porous medium (*e.g.* the membrane).

Traditional modelling approaches for HFB have been motivated by understanding mass transport of solutes (*e.g.* oxygen, glucose) and proteins in a single fibre unit, called the Krogh cylinder, which is assumed to be representative of the whole reactor. The concept of a Krogh cylinder was originally introduced to describe diffusion of oxygen from capillaries in human tissues,^50^ and is now a common approach in efforts to model HFBs.^51–55^ The model consists of a single fibre surrounded by an annulus of ECS; by assuming the each fibre is identical and that the fibres are arranged in a regular hexagonal pattern, analysis of transport in this single fibre unit is considered representative of that throughout the bundle.

One of the earliest examples of the Krogh cylinder approach is Apelblat *et al.* 1974,^56^ where the authors use coupled Navier–Stokes equations (lumen) and Darcy's law (membrane and ECS) to describe fluid transport. In Kelsey *et al.* (1990),^57^ Darcy's law was replaced with Navier–Stokes’ equations for a setup with no cells in the ECS. Bruining (1989)^58^ presented a general description of the hydrodynamics in hollow fibre devices. The scope of his analysis included different modes of operation (*e.g.*ECS ports open or shut) corresponding to various applications of HFBs. Starting from mass and momentum balance equations, Bruining obtained expressions for the hydrostatic pressure and bypass (fraction of fluid passing through the ECS). However, Bruining's analysis provides no information on local velocity profiles, which are essential to understand, control and predict the transport of nutrients or waste products by advection. A review of Krogh cylinder models for mass transport in HFBs for cell culture is presented in Brotherton and Chau (1996).^51^ Two further Krogh cylinder approaches modelled the ECS as a porous medium to mimic a densely packed cell population.^55,59^ Abdullah and Das (2007)^59^ consider the impact of multi-solute interactions by using the Maxwell–Stefan equations to describe diffusion. Numerical solutions of the full equations using finite-element methods are used to investigate the dependence of the nutrient concentration profiles on parameters such as the cell density, fluid flow rate and depth of the cell layer.

Various approaches by the current authors have focused on the development of equations and data to inform bioreactor design and operation. One example focused on water transport throughout a single module unit in the absence of cells;^60^ this was integrated with experimental data to determine the permeability of poly(vinyl alcohol) (PVA)–PLGA membranes to pure water transport and to define an operating equation that relates the input conditions (lumen flow rate, lumen exit pressure and bioreactor geometry) to the ratio of flow rates exiting either the lumen and ECS. An extension to this overlaid a Poiseuille flow model for fluid transport in the lumen with oxygen transport throughout the bioreactor; this took the form of an advection–diffusion, diffusion and diffusion–reaction equations in the lumen, membrane and ECS, respectively. The system of equations was reduced by exploiting the small aspect ratio of a fibre to give simplified relationships that describe the relationship between the oxygen concentration throughout the ECS, and geometrical (*e.g.* lumen length, ECS depth) and operational (*e.g.* flow rates and pressures) parameters that can be controlled by the user.^61^ This work was extended to account for more complex fluid flow environments in the membrane and ECS,^62^ as are encountered if the ECS ports are opened. Similar approaches have been tailored specifically to the use of HFB for BAL applications,^45^ and to explore zonation.^63^ Here the mathematical models described above are used to understand how parameters such as the fluid flow rate and lumen length can be exploited to setup controlled spatial gradients in oxygen concentration down the lumen length, thus mimicking zonation in a hepatic sinusoid.

Crucially, the mathematical modelling frameworks described above provide spatial information on the cellular environment that is costly to achieve experimentally, are cell-type specific, and provide relationships that enable a user to tune the bioreactor geometry and operation to the application under consideration. The further development of these frameworks provides the potential for sophisticated *in vitro* liver models that represent more accurately the physiological and toxicological scenario *in vivo*, by coupling spatial features such as zonation to hepatocyte function. With respect to predictive toxicology, hepatocyte function, in this case, is more concerned with the enzymes responsible for phase I and II drug metabolism and drug transporters, rather than the enzymes catalyzing more physiological processes such as ammonia and carbohydrate metabolism. Consequently, to build a stable mathematical modelling framework which is applicable to predictive toxicology, we require chemically, metabolically and toxicologically well characterised paradigm compounds with which to begin populating the framework. One of the most thoroughly characterised molecules in this respect is APAP. It is now pertinent to briefly review how APAP causes liver injury through metabolism and bioactivation, and how emerging *in vitro* models can help closely reflect the *in vivo* situation both physiologically and toxicologically.

## 5. Acetaminophen metabolism and bioactivation in emerging models

### 5a The normal process of acetaminophen-mediated liver injury

APAP is a very important and widely used tool to study mechanisms underlying the development of hepatotoxicity, injury progression and adaptation across *in vitro*, animal and human systems. It is widely recognised that oxidative metabolism of APAP to *N*-acetyl-*p*-benzo-quinoneimine (NAPQI) is crucial for the development of hepatic injury. Almost every subsequent event is still the subject of further research, debate and complexity.

The use of APAP and its associated metabolism, bioactivation and toxicity is a crucially important benchmark which can be used to cross-evaluate novel *in vitro* systems. The extensive APAP metabolism/toxicity literature allows for a thorough comparison of novel *in vitro* systems, and the extent to which they improve upon traditional cell cultures methods or re-capitulate the *in vivo* environment. Comprehensive multi-parametric comparisons with respect to the involvement of phase I and II drug metabolism (CYP2E1, CYP3A4, glucuronidation and sulfation), drug transporters,^64–66^ detoxification processes (transcription factors *e.g.* Nrf2), adaptive/regenerative and overt toxic responses during APAP exposure can be compared both quantitatively and semi-quantitatively. These in-depth ‘functional’ assessments are far more important and provide real-life data that can be used for *in vivo* comparison, than simple mRNA or protein determinations of whether a enzyme is present or whether a fluorescent probe substrate can be handled.

At therapeutic doses, APAP is metabolized predominantly by the phase II metabolic pathways of glucuronidation and sulfation. There is a species- and strain-dependent proportion of APAP which is metabolized by the phase I cytochrome P450 metabolic pathway to the reactive metabolite NAPQI, which is subsequently detoxified by conjugation with GSH.^67,68^ In higher dose situations, the sulfation pathway becomes saturated with depletion of the sulphate pool, the co-factors of glucuronidation become depleted, and hence a larger portion of APAP is metabolized through the phase I pathway. GSH levels are limited and once depleted below a critical level NAPQI is free to react with cellular macromolecules. After formation of significant quantities of NAPQI, the pathways culminating in cellular injury have been extensively investigated, but their contributions to the actual pathogenesis are more tentative.^38,67–69^ Based on the weight of evidence, NAPQI binds to various proteins and disrupts their function, leading to altered cellular function. However, there are likely to be other direct or indirect effects of NAPQI leading to cell death, such as alteration of cellular redox status or disruption of signalling pathways. Despite the multitude of cellular pathways that have been shown to play a role in APAP-induced hepatotoxicity, disruption of mitochondrial function is one of the key outcomes.^38,69–71^ After covalent binding and GSH depletion occur, APAP induces the mitochondrial permeability transition, which allows the leakage of mitochondrial constituents into the cytosol. After activation of the MPT, mitochondria swell, lose membrane potential, and exhibit decreased oxidative phosphorylation and ATP depletion. Finally, depending upon the species, strain and fed/fasting status there may be a window where some hepatic apoptosis occurs, which rapidly degenerates into necrosis.^72–74^

### 5b Acetaminophen metabolism and bioactivation in bioreactors

There are many different ways in which to assess bioreactor capability, this could be through the investigation of physiological parameters, such as cell viability, albumin production or urea detoxification. However, in order for hepatic bioreactors be useful in predicting chemical risk, the functionality of the drug metabolising enzymes needs to be an integration of drug clearance, metabolite formation and relative cytotoxicity *vs.* static cultures. Many publications use cytochrome P450 transcript or protein level as a marker of cell functionality, which although helpful, are not assessments of enzyme function. For example, freshly isolated hepatocytes will have a full message and protein complement, yet are likely to have rapidly decreasing functional cytochrome P450 capability due to enzyme inhibition by reactive oxygen and nitrogen species.^75,76^

In order to assess the relative merits of different bioreactor formats, it is necessary to utilise paradigm compounds that have very well characterised *in vitro* pharmacokinetics and toxicity profiles, such as APAP.

Allen *et al.* (2005)^35^ employed a flat-plate bioreactor to impose physiologic gradients over phenotypically stable hepatocytes and evaluate spatial variations of CYP expression and toxicity. Perfusion of APAP resulted in a shift in the dose response such that 20 mM was completely toxic in bioreactor cultures as compared to 40 mM in static cultures. Also, perfusion cultures at 15 mM demonstrated a toxicity pattern similar to the centrilobular localization seen *in vivo*. This *in vitro* zonal toxicity provides insight into the deleterious effects of APAP and the factors that contribute to spatial toxicity which would not be observed in conventional culture models. Toxic effects in this study are likely due to the depletion of glutathione, which provides protective inactivation of NAPQI. Though centrilobular localization of APAP toxicity *in vivo* has been attributed to local expression of CYP isoenzymes 2E1 and 3A, reduced oxygen availability in centrilobular regions may also contribute by depleting ATP and glutathione, or increasing damage by reactive species. A combination of these factors likely resulted in the regional toxicity observed in reactor cultures under dynamic oxygen gradients. Demonstration of zonal toxicity *in vitro* allows decoupling of the effects of CYP bioactivation and glutathione levels on acute APAP toxicity.^35^

Hepatocyte sandwich perfusion culture can further improve long-term liver specific functions *in vitro*, due to the improved transport of oxygen and nutrients to the cell surface and effective waste removal from cellular local environment. Perfusion bioreactors have been developed based on conventional sandwich culture which has a hepatocyte monolayer overlaid by a collagen gel layer 24 h after seeding.^77^ However, the perfusion flow would introduce the hepatocyte culture to the effect of fluid-induced shear stress not typically encountered by the cells in natural liver where hepatocytes are shielded by a layer of sinusoidal endothelial cells from the direct shear of the blood flow. High shear stress in perfusion culture could be detrimental to hepatocyte viability and *in vitro* functions. In addition, the integrity of the top collagen layer in direct contact with media flow may be compromised by the long-term perfusion that leads to the degeneration of sandwich matrix and, subsequently, the variation in mass transport of drug access during drug testing.^77^

Xia *et al.* (2009)^77^ have developed a laminar-flow perfusion bioreactor for immediate-overlay sandwich culture that minimizes shear stress and preserves the mass transport consistency. The cultured hepatocytes exhibited restored cell polarity, biliary excretion and maintenance of metabolic functions for two weeks. Liver specific functions of hepatocytes, such as the phase I drug metabolic function (*e.g.* EROD activity) are reported to be maintained for up to 15 days. The perfusion culture exhibited a higher sensitivity to APAP-induced toxicity than the static culture on both day 7 and day 14, attributable to the well-maintained drug metabolic functions of the sandwich perfusion culture. Approximately 60% of cell death was observed in the perfusion culture treated with 25 mM of APAP for 24 h. Furthermore, the cell viability in the perfusion culture after APAP treatment on day 7 and day 14 was similar. In contrast, static culture treated with APAP on day 7 and day 14 produced highly variable cell viability results.

More recently, Prot *et al.* (2011),^78^ have attempted to combine enzymatic functionality with proteomic and transcriptomic assessment of their flat-bed, HepG2-populated bioreactor. Classical studies demonstrating the hepatotoxic effect of APAP performed *in vitro* show that cytotoxicity is observed for a concentration of APAP ranging up to 5 to 20 mM with interspecies differences. In this case, APAP led to an EC50 at a 1 mM concentration for 72 h of contact only in the microfluidic biochip configuration.^78^ This result is in accordance with the toxic plasma level observed in humans and which ranges between 1 and 2 mM. They did not take into account the protein binding to APAP. Although APAP has a weak affinity for plasma proteins (<20%), the protein content between blood and culture are different. Consequently, the protein binding quantification will be necessary to fully confirm that the microfluidic biochip condition is a more physiological situation.^78^

This is a particularly encouraging report, as HepG2 cells in particular are not renowned for their metabolic capability.^79^ Other hepatic cell lines have been reported to have greater metabolic functionality, however, this is only when treated with proprietary dimethyl sulphoxide-containing ‘induction-media’.^79^ The recreation of more physiological environment for hepatic cell lines, including flow, oxygen levels, a 3D scaffold, the presence of endothelial cells appears to allow much improved functionality over static cultures. However, the relative importance of each of these parameters should be investigated in such a way as to define whether it is crucial for enhancing metabolism in chemical risk assessment *in vitro*.

This process will be time consuming and costly to conduct using experiments alone. In the next Section we discuss the use of mathematical models to inform cellular pharmacokinetics, using APAP as an example case. Compared to the mathematical models for transport processes within a bioreactor, here we focus on the pharmacokinetic processes within an individual cell. Such models enable insight to be gained into the relative importance of the range of metabolic processes occurring, can be used to test hypothesis *in silico*, and also can inform experimental work, thus providing a useful tool to complement existing experimental techniques.

## 6. Modelling cellular pharmacokinetics: paracetamol as a case study

The Hill model is a fundamental analytical component for many pharmacokinetic–pharmacodynamic mathematical models.^80^ It is a mathematical function that relates the uptake rate of a compound to the underlying compound concentration, and has insightful explanatory properties in the cases of a physicochemical equilibrium:^81^ for example, in helping to identify specific aspects of a drug; or comparing drug effects; or predicting the state of the system under variable conditions; or identifying an effective dosage regime (see ref. 81 for a review). Even beyond the equilibrium, the Hill equation has been shown to be of use, for example see Zhi *et al.*'s^82^ pharmacodynamics model for antibiotic treatment of microorganisms. Analogies of the Hill function to cumulative probability curves have also been drawn and the Hill equation has been used to build probabilistic models, for example see Rougier *et al.* ^83^ in which the Hill equation is used as an expression of a cumulative probability distribution for the occurrence of aminoglycoside nephrotoxicity. As Goutelle explains,^81^ the probabilistic extension of the Hill equation is an exciting prospect as it may permit estimation of the probability of an adverse reaction and can therefore potentially be used to make important clinical decisions.

Studying dose response models has been found to be useful in determining safe and hazardous levels and dosages for drugs. A dose-response curve is a simple graph relating the concentration of a compound to a given response (*e.g.* physiological, biochemical *etc.*). Analysis of dose response curves is typically performed using regression methods; the curves are typically fitted to the Hill equation (also known as the four parameter logistic fit). Over the years, a large number of studies have used Hill equations for the determination of drug potency, but there are a number issues related with this method. Prinz^84^ points out that Hill coefficients may give insight into the degree of binding cooperativity but cannot distinguish between competitive, non-competitive or *ortho*-, iso-, or allosteric mechanisms. The Hill function is clearly a simplification and it is likely that cell responses to toxic chemical compounds may not follow a simple logistic curve. Variation in the curve shape, however, has been explored by Levasseur.^85^

Levasseur^85^ has fit a number of models to data for seven anticancer agents against both parental and drug-resistant cell lines with iteratively reweighted non-linear regression. The key focus of this work was to model simultaneously both exposure time and drug concentration, rather than the typical approach at the time of this study which was to focus on concentration effect curves (see for example,^86^). The models used in their fitting take the form of either single, double or triple hill concentration effect curves, to represent either single cell population sensitivity or two or three populations of cells with different sensitivities and heterogeneities of drug responses. These types of models are useful for quantitative assessment, for example in assessing a growth-inhibitory effect of agents as a function of concentration and exposure time, but they can offer limited insight into underlying physicochemical mechanisms as the Hill parameters in this case have no physical meaning.

Some previous cellular pharmacodynamic models have included cellular pharmacokinetics, for example see.^87–90^ The^87,88^ models are discussed below. The model by Lankelma *et al.* ^89^ incorporates two intracellular components with cell kill from the anticancer agent doxorubicin taken to be a function of compartmental concentration. In Lobo and Balthasar,^90^ delays are used to implicitly account for cellular uptake and binding for methotrexate.

A pharmacokinetic–pharmacodynamic model has been developed by Kareh *et al.* ^87^ for the cellular pharmacology of paclitaxel with a critical dependency on the cell cycle – in particular cell cycle checkpoints. Here, cell death is assumed to occur when a threshold level of a lethal compound is exceeded once the cell is at a particular cell-cycle checkpoint. An assumed lognormal probability function for cell survival fraction accounts for cell population heterogeneity. The asynchrony of cell-cycle stages within the cell population is simplified by defining cell damage as the average over all the cells in terms of a maximum value of the concentration of the lethal compound. Experimental observations relating extracellular and intracellular concentrations of paclitaxel in human breast MCF cell monolayers^91^ has been used to determine the cellular pharmacokinetics for paclitaxel. This assumes equilibrium conditions with depletion, protein binding *etc.* ignored. Multiple damage mechanisms are taken into account corresponding to damage incurring at G1 and G2M phases of the cell cycle. Additive, where the total damage is represented as an exponent weighted sum, and independent damage models are compared. The cellular pharmacokinetics are based on experimental uptake rates^92^ but, rather than model the detailed pharmacokinetics, pharmacological terms are chosen to represent how the intracellular concentration of the lethal compound depends on the extracellular concentration. These terms are additionally assumed to decay exponentially over time to represent DNA repair timescales.

Another model for paclitaxel is that by Kuh.^91^ This model was based on a deterministic pharmacokinetic system to describe paclitaxel uptake, binding and efflux from cells. The key aim of the model was to predict the intracellular concentration of the drug. Ordinary differential equations were used to describe the changes over time of the concentrations of extra- and intracellular paclitaxel, total (free plus bound) drug in media and cells, and free drug in media and cells. There was an explicit consideration of cell volume, which was taken to be an exponentially decaying/increasing function of time due to cell cytotoxicity/cell proliferation – which of these that occurs depends on different drug concentrations. Extracellular drug binding was assumed to occur *via* Michaelis–Menten kinetics with a constant binding rate. Similar saturating binding kinetics are assumed to also occur intracellularly but, in this case, the binding rate was assumed to increase linearly with time. This was based on experimental observations showing a linear enhancement of tublin concentration over time with paclitaxel treatment. An additional linear intracellular binding term was also included to take into account non-saturable binding. The resulting mass balance equations are solved numerically and a parameter sensitivity analysis is performed to explore effects on intracellular drug accumulation.

In the work of Rougier *et al.*,^83^ the area under the time concentration curve (AUC) of amikacine serum concentrations was used to calculate the probability of occurrence of nephrotoxicity. The AUC is a commonly measured pharmacokinetic metric used to estimate exposure effects to a drug (*e.g.* see ref. 89 which notes that cell uptake and cytotoxicity of doxorubicin depends on the shape of the AUC). However, not all studies support the idea that cell kill is just a function of the AUC (see *e.g.* ^85,93^) but note that many of these studies do not account for transport of drug into the cells which is suggested be an important factor.^88^ El-Kareh investigates the effectiveness of the AUC-dependent cytotoxicology measure to test anti-tumour effects to doxorubicin in ref. 88. The model is similar to that of^87^ but the principle focus was on the cellular uptake and cytotoxicity of the drug. Cell cycle effects were also neglected, a Hill-type dependency of cell survival on drug induced damage was assumed, cell damage was taken to be an additive sum of extracellular and intracellular effects, and the dependence of cell kill was on peak values of concentration rather than on the AUC. El-Kareh^88^ reports an overall better fit to *in vitro* cytotoxicity data than compared to previous AUC-dependent models.

The use of the Hill function, however, is limited in that it fails to account for underlying physicochemical reactions. Beyond single or positive cooperative binding interactions the Hill equation is somewhat of a compromise and, given that often in pharmacology, binding events are not restricted to single drug-receptor interactions but are instead more likely to be aggregated measures of drug effects on a large number of receptors,^81^ the Hill equation parameters have very little physical meaning. We propose that a mechanistic model that describes the physiochemical interaction at the receptor level and incorporates explicitly drug transport across the cell membrane and includes extra- and intracellular drug kinetics will best facilitate the quantitative assessment of drug metabolism. Cellular uptake data (see for example^94^ for data on doxorubicin) shows that intracellular drug levels often take on the order of hours to equilibrate with those extracellularly, suggesting that it is important to keep track of the non-equilibrium transient kinetics of uptake and cytotoxicity and therefore we propose that a non-equilibrium model is often most appropriate. We will now describe an example of a modelling framework that takes into account these factors and we use APAP as an example compound.

### 6a Case study: a mathematical model of paracetamol metabolism

The aim of this section is to illustrate how a mathematical model can be derived to describe, in simple terms, the metabolism of APAP in hepatocytes. A model that is very detailed, *e.g.* one that accounts explicitly for all the genes, proteins, enzymes *etc.* involved in a metabolic pathway, will normally be far too complex to be analysed mathematically and the only effective means of progress is through simulation using computational methods. However, such models will have many parameters (*e.g.* rate constants, initial concentrations) and consequently there is a huge degree of freedom influencing model outcome, not least because the values of many of these parameters will be unknown. Because of the degrees of freedom, a simulation giving good quantitative agreement with data does not necessarily mean we have an accurately parameterised model, as changing the value of one parameter may be compensated by changing another to obtain equally good agreement. In such cases, simulating the system in a perturbed scenario has limited predictive worth, because, if the chosen set of parameters is incorrect, the predicted results may be wildly different to that of reality. In contrast, a simple model, incorporating only the “major” factors in the system, has the advantage of being less complex, having fewer parameters and having a much greater range of mathematical methods available for its study. Such analytical methods provide a deeper understanding of the model, which in turn provides a deeper understanding in the role of each of the mechanisms in the system being modelled and, as is hoped, will offer new insights into the biology. However, in proposing simple models there is a cost in the exclusion of presumed “minor” mechanisms, which could mean a loss of quantitative resolution in the model results. The craft of the modeller is to derive a model that is balanced in detail and simplicity, which is inevitably a cyclic process working closely with the experimentalists. The relatively simple model presented below is an initial venture into modelling APAP metabolism in hepatocytes. The model has yet to be analysed and parameterised, but it serves to illustrate how a metabolic pathway can be described using a fairly simple mathematical model.

**Fig. 4:**
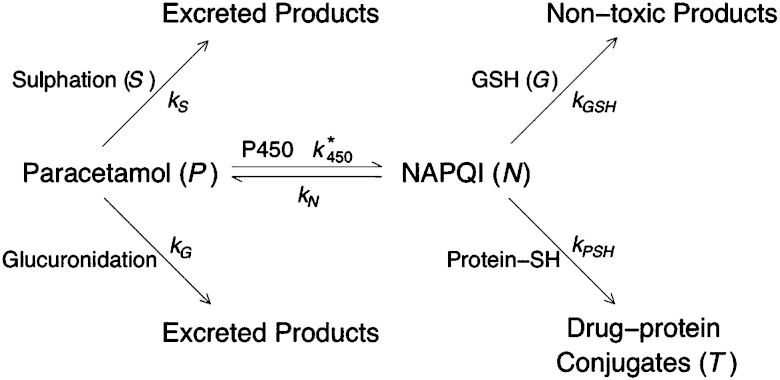
Diagram summarising the paracetamol metabolism pathway in hepatocytes. The italicised capital letters indicate the model variables and the subscripted “*k*”s are the rate constants for the particular path.

An important step in model development is to establish the mechanisms that are most likely to be the most important in governing the process. To this end, a very useful starting point is to put together a pathway diagram of the process, which can be clearly understood by both the modellers and experimentalists. Once such a pathway is in place then it is usually straightforward to write down a system of equations describing the evolution of the components by, for example, using mass action laws (Fig. 4 shows the pathway diagram used in the model described below). In our preliminary model, we assume that all the components of the APAP metabolic pathway are well mixed in the cell, or at least well mixed in the local site of the cell at which these processes are taking place; this assumption means that we can avoid the added complication of accounting explicitly the spatial variations of the components. This means that our main variables only change in time and the evolution of them can be described in terms of ordinary differential equations.

We assume that APAP (concentration *P*) is introduced into a cell with initial concentration P*0; this will of course vary with dosage and also will be dependent on the location of the cell along the hepatic sinusoid (we note that parameters that depend on location will be denoted with a *). From Fig. 4 we observe that APAP can be converted to excretory products, *via* sulphation and glucuronidation, or react with P450 to form NAPQI (concentration *N*). In our first approximation, it is assumed that P450 and the co-factors involved in glucuronidation are present at such high concentrations as to be little effected by the metabolism of the drug, *i.e.* their concentrations remain approximately constant throughout. For co-factors involved in sulphation, the story is expected to be different, in that their concentrations do change during this process; consequently, we need to track the concentration of these compound(s) (concentration *S*). We will assume also that NAPQI can spontaneously breakdown, releasing APAP back into the system. Combining all these assumptions we write
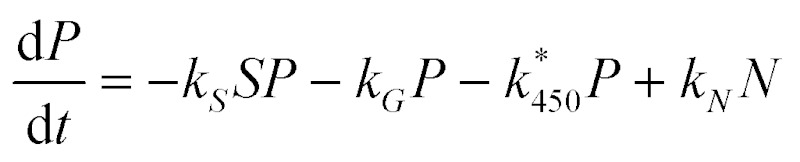


The concentration of P450 is dependent on location in a liver lobule, hence the * reaction rate constant k*450.

In the absence of drug, we assume there is a natural turnover of the sulphation co-factors, being produced constitutively at a rate *β*_*S*_ and naturally breaks down with decay rate constant *δ*_*S*_. Combining this with the removal by sulphation, leads to the equation
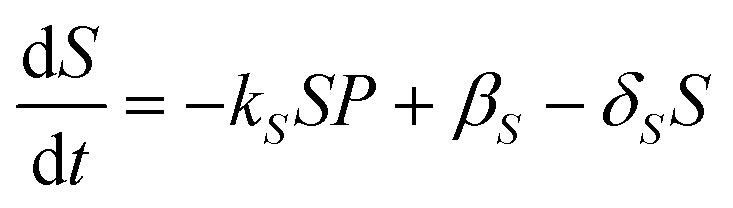


As discussed above NAPQI accumulates by the (reversible) reaction of APAP with P450. It is assumed that it can combine with GSH (concentration *G*) to form harmless products or, less desirable, with proteins-SH to form drug–protein conjugates (concentration *T*). There are numerous types of proteins-SH that will react with NAPQI and we will, for simplicity, lump all the protein-SH types together. Furthermore, we assume that there is an inexhaustible quantity of them, and describe the overall reaction along the toxin branch as a simple NAPQI decay term. The combination of these assumptions yields
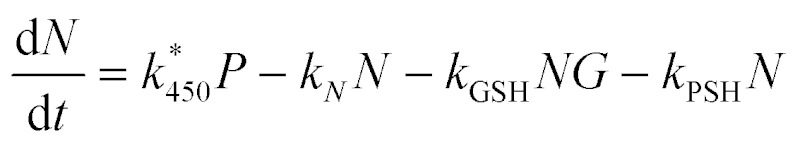


This equation contains the key balance between the success and failure of the hepatocytes dealing with the build-up NAPQI effectively. Initially, GSH will be present in “large quantities”, which means that the breakdown of NAPQI will be predominantly along the branch towards non-toxic products. Mathematically, this says that the quantity *k*_GSH_ *G* is much larger than *k*_PSH_. However, as *G* is being consumed and eventually exhausted, then this relationship will switch and the branch towards drug–protein conjugate production will dominate.

As with the sulphation compounds, we assume in the absence of APAP GSH is produced at a constitutive level (at rate β*G) and decays naturally (with decay rate constant *δ*_*G*_), so that the concentration of GSH in healthy liver cells will be β*G/*δ*_*G*_. We note that GSH levels do vary in the lobule, which we have assumed to be due to differences in production rate. Combining this with reaction of NAPQI yields
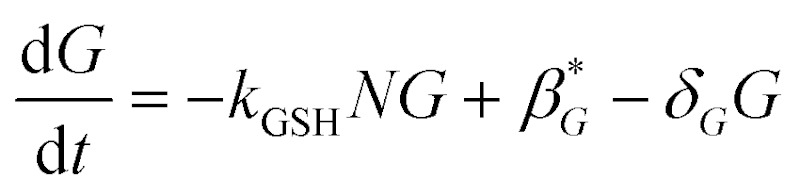


The build up of drug–protein conjugate, *T*, will reflect on the amount of damage the APAP dose will do to the cell. It is only produced by the reaction between NAPQI and protein-SH and we write
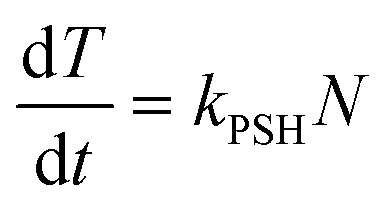


We have not considered any further reactions involving the drug–protein conjugate, and the variable *T* represents an accumulated total concentration of toxic species, rather than the amount of drug–protein conjugate at a particular time point; we could add a decay term to this equation as a simple way to describe these reactions. In order to complete the model we need to introduce an initial state, say at *t* = 0; we assume that the hepatocyte starts off as being healthy and is dosed with APAP, and write *P*(0) = P*0, *S*(0) = *β*_*S*_/*δ*_*S*_, *N*(0) = 0, *G*(0) = β*G/*δ*_*G*_ and *T*(0) = 0.

This relatively simple model consists of ten reaction rate constants. Parameterisation of models such as these always poses a challenge and an extensive trawl through the experimental literature will be required to find as many suitable estimates as possible. This process will help to identify deficiencies in data and motivate further experimentation. Moreover, the model is simple enough to study using a range of mathematical techniques, in which we may be able to establish a full understanding of how the parameters affect the predicted outcome, for example, deriving a simple formula that predicts the extent of damage given a dose of the drug.

## 7. Use of population IVIVE-PBPK approaches in assessing drug/chemical toxicity

Given the wealth of mathematical and experimental models now available to explore hepatocyte function, a key question is how to relate this *in vitro* information to the human physiological environment *in vivo*. One solution to this is the use of *in vitro*–*in vivo* extrapolation (IVIVE) techniques coupled with physiologically based pharmacokinetic models, and this approach is now routinely used as a tool to simulate and model the exposure of drugs following dosing to humans and pre-clinical species.^95–100^ The combination of these approaches allows the systemic concentration and tissue levels of the drug/chemical of interest to be simulated. The concentrations in the various tissues can be coupled to pharmacodynamic models to simulate pharmacological and toxicological effects of the drug/chemical in addition to the pharmacokinetics of compounds.

The basic principles of IVIVE include making *in vitro* measurements of the rate of drug/chemical metabolism, measuring (or predicting) the binding of the drug to plasma proteins and the non-specific binding within the experimental *in vitro* system and a knowledge of the blood : plasma ratio of the compound. Using physiological scaling factors such as the amount of microsomal protein or number of cells per gram tissue together with the weight of the organ the *in vitro* metabolism data can be scaled up to give an estimate of the intrinsic clearance of the drug/chemical in the whole organ. By incorporating this information into a model accounting for blood flow through the organ the clearance of the drug in the organ can be predicted.^101^

The second characteristic of the drug/chemical that needs to be understood to use PBPK approaches is the distribution of the drug/chemical into the tissues of the body. Traditionally, this information could only be generated by performing studies in animals where compounds were administered (often by infusion to steady state) then animals were euthanized and plasma and tissues removed and the drug concentrations measured. This allowed calculation of the tissue : plasma ratio in each organ.^95^ Although many PBPK models have been constructed using this approach (*e.g.* ^102^) the experimental work needed to construct a PBPK model was laborious, time consuming and required extensive experimentation in animals. Another limitation was that the experiments needed to be performed for each compound of interest and as such was not suited to the rapid evaluation of large numbers of compounds needed in a drug discovery environment. In recent years *in silico* methods have been developed that allow the tissue : plasma ratios for compounds to be predicted using a knowledge of the compounds physicochemical properties.^103–108^ This has revolutionised the use of PBPK models within the Pharmaceutical industry as for a given compound routinely measured physicochemical data (log *D*, p*K*_a_,) and *in vitro* data (plasma protein binding; blood : plasma ratio) can be used to predict the distribution of drugs into tissues. By combining the IVIVE approaches to predict CL and *in silico* approaches to predict tissue distribution the disposition of a drug/chemical can be simulated. Furthermore, if information on the variability of each of the parameters used in the model is known then it is possible to apply IVIVE-PBPK approaches to predict the exposure (and effects) of drugs and chemicals in populations rather than in an “average” individual.^101^

As exemplified below this population IVIVE-PBPK approach has been recently applied to understand the toxicological risk posed by some drugs and chemicals and some aspects of the application of population PBPK approaches in human risk assessment have been recently reviewed.^109^

### Example 1: linking *in vitro* toxicology data with population IVIVE approaches

The 2007 report Toxicity Testing in the 21st Century: A Vision and a Strategy argued for a change in the way that toxicity testing of environmental agents was performed with a move away from *in vivo* animal testing towards toxicity testing based on understanding pathway perturbations observed in human cells *in vitro* with tests being done in a high throughput format. This high throughput *in vitro* testing paradigm is now being utilised in both the Toxcast (US EPA) and Tox 21 (NIH) programs.^110,111^

Although the *in vitro* assays can give a measure of the hazard posed by individual chemicals, prioritisation of chemicals based only on the toxic concentrations in *in vitro* assays without considering the impact of bioavailability, clearance, and exposure could over- or under-estimate the potential risk of these chemicals to human health.^112^

A number of studies have been conducted to try and link IVIVE-PBPK approaches with high throughput toxicity testing results to estimate the exposure/dose that would lead to toxicity in the more susceptible individuals in a simulated human exposure. The information from the population IVIVE-PBPK approach is compared with an estimated of the actual exposure in human populations. By comparing the window between simulated and actual exposure the compounds where toxicity is most likely can be selected for further, more definitive testing.^27^

This strategy was exemplified in a study by Rotroff *et al.* (2010).^113^ In this study metabolic clearance was measured in primary human cryopreserved hepatocytes and plasma protein binding was measured *in vitro* for 35 Toxcast chemicals. The metabolism and binding data were used as inputs in the Simcyp population-based IVIVE programme.^114^ Renal clearance was estimated from the glomerular filtration rate and protein binding and absorption was assumed to be 100%. From this the human equivalent dose to produce a steady state *in vivo* concentration equivalent to the concentration causing 50% of the maximum activity *in vitro* or the lowest effective concentration values from the Toxcast data could be calculated.^113^ These doses were then compared with chronic aggregate human oral exposures (where known) to see if biological activity and exposure overlapped. This occurred for only 2 compounds which would not have been at the top of the list based only on *in vitro* potency criteria.

In a follow-up study Wetmore *et al.* ^29^ extended the *in vitro* measurements to a total of 239 compounds. In addition to hepatocyte stability and plasma protein binding in this study Caco-2 permeability data and blood : plasma ratio data was also generated for some compounds. For 18 (9.9%) chemicals, where data was available, the estimated (from IVIVE-PBPK) dose that would exert a biological effect in the population was equivalent to or less than the highest exposure estimates in the US population. The biological effects perturbed included cell growth kinetic effects, alterations in cytokine and drug metabolising enzyme/transporter expression and CYP inhibition, also affected were prostaglandin E receptor and urokinase-type plasminogen activator, which were both down regulated. Judson *et al.*,^111^ have also reported case studies (bisphenol A estrogenicity and for conazole CAR/PXR activation) where the ability of the population IVIVE-PBPK approaches to predict doses that would perturb the biological pathway was compared with the doses derived from animal toxicology studies.

Population IVIVE-PBPK approaches are also being utilised in an EU funded framework 7 project (predict-IV). (http://www.predict-iv.toxi.uni-wuerzburg.de). The overall aim of the project is to develop strategies that improve safety assessments in the late discovery-early development stage of the drug discovery process. To do this a combination of non-animal based *in vitro* test systems (including high content screening), mechanistic toxicology approaches (including toxicogenomics and metabolomics analysis) together with *in silico* modelling approaches are being utilised.

### Example 2: using population IVIVE-PBPK approaches to define the window between efficacious dose and the doses where adverse effect are expected to occur

A second utility of population IVIVE-PBPK modelling was described by Shaffer *et al.* (BDD 2012). During a discovery project looking to nominate an α-amino-3-hydroxy-5-methyl-4-isoxazolepropionic acid (AMPA) receptor modulator for advancement into human trials, as a possible treatment for CNS disorders, three potential compounds were identified. Using available *in vitro* data on the metabolism and binding of the compounds in humans, together with information on the potency of the compounds and the plasma levels at which the compounds caused beneficial and adverse events in pre-clinical species the following strategy was employed. Simulations of the pharmacokinetics and expected population variability of the three compounds were made using the Simcyp simulator. Simulations were conducted to define the doses at which the plasma concentrations achieved the estimated efficacious concentration. The maximal concentrations (*C*_max_) in these simulations were also compared to the plasma levels at which adverse events were anticipated to occur. By doing this it was possible to rank the compounds and to identify the compound that was predicted to have the biggest window between expected efficacious dose and the dose at which adverse events were expected to occur in humans.

### Example 3: using population IVIVE-PBPK-PD approaches

Attempts have also been made to use concentrations from IVIVE-PBPK simulations as input (driving) concentrations for more sophisticated pharmacodynamic models. An example is combining the concentrations of basic drugs in the plasma and heart with *in silico* population models of the cardiac myocyte (Polak *et al.*, Toxicology Mechanisms and Methods, 2012; 22, 31). This approach then allows the relationship between drug concentration and changes in cardiac action potential to be modelled by considering for instance the pharmacological consequences of inhibition of the sodium and potassium channels in the heart.^115^

As greater application of IVIVE-PBPK approaches to tackle toxicological problems are made there is a great opportunity to use the models to help reduce the needs for *in vivo* testing of chemicals in animals, and also to make greater use of these approaches to predict the likely toxicity of chemicals in humans. For instance Tonnelier *et al.*,^116^ have examined the ability of IVIVE-PBPK approaches to predict human bioaccumulation of environmental chemicals whilst Tan *et al.*,^117^ have discussed the challenges with extending these approaches to look at the interaction between and subsequent toxicological outcome when chemical mixtures are dosed rather than individual chemicals.

Although the PBPK models currently used provide useful information further refinements of the models have been suggested including alternative representations of the tissue compartments^118^ and it is likely that to understand some of the more complex aspects of drug/chemical toxicology more detailed models that can account for differences in zonation across the liver, specific sub-populations of cells that are at risk of toxicity etc will be needed to accurately predict the relationship between tissue concentration and toxicity in susceptible individuals. In addition the challenges that need to be resolved to allow the use of IVIVE and PBPK approaches in formal human health risk assessment have been recently discussed.^119^ Whilst the combination of high throughput *in vitro* toxicity data with population IVIVE approaches is showing great promise particularly as a tool to prioritise compounds for further consideration there are some limitations of the high throughput screening approach. These include the fact that most of the cell types used in high throughput assays lack the ability to catalyse chemical biotransformation (particularly at a quantitatively relevant rate) and as such often will only allow information on parent compound (not more or less toxic metabolites) to be made. In addition using only a single cell type as is often done then a number of paracrine signalling pathways may be missing and in order to restore these, a mixture of cell types, appropriate extracellular matrices and three dimensional geometries may be needed. Both of these limitations outline the need for more complex *in vitro* systems that can be used to generate detailed mechanistic understanding of the toxicological risk of compounds identified in the initial screening approach.

## 8. Adding value through multidisciplinary and 3Rs approaches

The concept of the replacement, reduction and refinement of animals in research (the 3Rs) was first set out in 1959.^120^ These principles, which provide a scientific and ethical framework for the humane use of animals in research, have long been embedded in UK and European legislation on the use of animals in scientific procedures. However, for a long time they have to some extent been seen as a marginal activity, and it is only now that they are being recognised more widely as a legitimate scientific goal that can provide benefits not just in terms of animal welfare but also providing more predictive or efficient tools that benefit science and business.

Within the field of toxicology, studies have traditionally been conducted in animals at high doses with default methods used to extrapolate to low level exposures in human populations. However, there is growing interest in developing new approaches that could revolutionise how toxicity testing is conducted. In addition to animal welfare considerations, there are a number of economic, legislative and scientific drivers for this *e.g.*: (i) *in vivo* toxicity studies are expensive and time consuming, which limits their practicality in large-scale chemical testing programmes such as REACH; (ii) recent changes to the European Cosmetics Directive prescribe a ban on animal testing and the marketing of products containing ingredients tested on animals; (iii) the animal data can be difficult to interpret in terms of predicting potential effects in humans.

In 2007 the US National Research Council (NRC) published a landmark report on ‘Toxicity Testing in the 21st Century: A Vision and a Strategy’.^121,122^ The NRC was tasked with considering how toxicity testing could incorporate recent advances in science and technology in order to generate more relevant data for risk assessment, expand the number of chemicals of concern that could be assessed, and reduce the time, money and number of animals involved. The report sees a future in which routine toxicity testing would be conducted in primary human cells, human tissue surrogates, or human cell lines *in vitro* by evaluating cellular responses in a suite of toxicity pathway assays. These tools would enable risk assessors to focus on predicting exposures that are expected to be without adverse consequences, rather than making predictions on the incidence of specific adverse responses in human populations.

Implementing this Vision will require a major research effort involving scientists from a broad range of disciplines and sectors. Key elements to its realisation include identification and understanding of toxicity pathways, plus the development of systems to understand exposure parameters *in vitro* and their extrapolation to inform safe *in vivo* exposure/in use scenarios. A number of research efforts are currently ongoing, with some taking a case study approach with a specific toxicity pathway, and others applying a more unbiased approach seeking to design convenient *in vitro* systems that would provide information on as much of the biological response landscape as possible.^27,123,124^

The UK National Centre for the Replacement, Refinement and Reduction of Animals in Research (NC3Rs) works closely with scientists in industry, academia and government organisations to identify opportunities to minimise animal use and/or improve welfare, using the 3Rs as a framework to drive innovation. In 2011, the Centre launched CRACK IT (; http://www.crackit.org.uk), a new open innovation programme designed to accelerate the application of the 3Rs by fostering and funding multidisciplinary collaborations that broaden the expertise involved. A central component of CRACK IT is a new challenge-led research competition, CRACK IT Challenges. For this competition, the NC3Rs works closely with companies from the pharmaceutical, chemical, agrochemical and consumer product industries to identify global business challenges relating to the use of animals. Challenges are supported by funding from the NC3Rs and in-kind contributions from industry such as data, compounds and equipment.

The 2011 CRACK IT Challenges competition included a Challenge seeking to stimulate further research to explore a pathways approach to safety risk assessment. The research award was made to the authors of the present paper, on the strength of the truly multidisciplinary approach proposed; integrating new tissue engineered liver models based on human cells with mathematical modelling in order to promote liver zonation and explore effects on biological pathways associated with liver toxicity. The various approaches will ultimately be combined with the aim of extrapolating from *in vitro* to make decisions around safety in humans. This CRACK IT project will, if successful, provide proof of principle that the pathways approach proposed as a means of replacing animals in toxicology studies can be successfully employed. This project is complementary to and collaborative with the established Hamner DILIsim project and the new Innovative Medicines Initiative project “Mechanism-Based Integrated Systems for the Prediction of Drug-Induced Liver Injury” (MIP-DILI).

## 9. Summary – predicting systemic toxicity with a combined approach

The need for better predictive models for systemic toxicity is clear. There needs to be an appreciation that organ toxicity is a spectrum of biological processes from effects on single pathways in single cells (*e.g.* hepatocytes) to complex, time-dependent multi-cellular processes (*e.g.* innate immune-mediated injury). Cell injury can result from exposure to chemicals, is not a single biological process and involves interplay between chemical and biological factors. Key technology is the production and application of zonated hepatic bioreactors, enabling culture with enhanced physiological and toxicological representation. Bioreactors have been shown to be superior over collagen/matrigel adherent cell culture for facilitating the development of normal cellular function, *e.g.* resynthesis of cytochrome P450 enzymes post-isolation. Bioreactors need to be utilized which reproduce physiological (oxygen/glucose consumption, urea/albumin production) and functional (CYP-mediated metabolism) hepatic zonation. Future approaches should be underpinned and informed by a strong mathematical modelling/systems biology approach that will form a data framework consisting of circulating drug and metabolite levels, tissue/cellular burden of metabolites, glutathione and covalent binding levels, adaptive responses (Nrf2/NFkB nuclear translocation), apoptosis and necrosis biomarkers, this would allow more accurate *in vitro* to *in vivo* extrapolation to both animals and man. Chemical engineering has the tools, in the form of ‘unit operations’, *i.e.* linked but discrete events, to describe chemical processes, in this case systemic toxicology. The units in this system are the tissue bioreactors, focusing on the liver unit, yet expandable to other tissues. Mathematical modelling should be designed to both provide a framework with which to perform gap analysis, preventing the duplication of experiments, but also to mimic the structure of the experimental work allowing use of cell-scale modelling to understand metabolite interactions, extrapolation of the cell-scale behaviour to a liver tissue bioreactor and incorporation of co-culture/multiple bioreactor units.
